# Has COVID‐19 affected dementia diagnosis rates in England?

**DOI:** 10.1002/gps.5976

**Published:** 2023-07-22

**Authors:** Jemma Hazan, Kathy Y. Liu, Jeremy D. Isaacs, Alistair Burns, Robert Howard

**Affiliations:** ^1^ Division of Psychiatry University College London London UK; ^2^ St George's University Hospitals NHS Foundation Trust London UK; ^3^ St George's, University of London London UK; ^4^ University of Manchester Manchester UK

**Keywords:** assessment, COVID‐19, dementia, diagnosis, memory service

## Abstract

**Background:**

The COVID‐19 pandemic impacted on the provision of care and routine activity of all National Health Service (NHS) services. While General Practitioner referrals to memory services in England have returned to pre‐pandemic levels, the estimated dementia diagnosis rate (DDR) fell by 5.4% between March 2020 and February 2023.

**Methods:**

In this paper we explore whether this reduction is accurate or is an artefact of the way the NHS collects data.

**Results:**

We explore the processes that may have affected national dementia diagnosis rates during and following the COVID‐19 pandemic.

**Conclusions:**

We discuss what action could be taken to improve the DDR in the future.

## Key points

- Despite General Practitioner (GP) referrals to memory services in England returning to levels seen before the pandemic, there was a decline of 5.4% in the estimated dementia diagnosis rate (DDR) from March 2020 to February 2023.
- This paper explores the factors which may have affected the national DDR reduction. These include a backlog in dementia referrals, a reduction in coding of diagnoses, or a decrease in true dementia prevalence secondary to excess COVID‐19 deaths which has yet to be reflected in the DDR denominator.
- Further work is suggested to accurately capture dementia prevalence in the United Kingdom (UK). These include an up‐to‐date multicentre population‐based cohort study and adjusting the DDR denominator for factors known to affect dementia susceptibility such as deprivation, rurality, and ethnicity.

## 1 INTRODUCTION

The estimated number of people with dementia in the UK is 850,000.^1^ Following the 2012 “Prime Minister's Challenge on dementia,^2^ which prioritised increased diagnosis rates, the National Health Service (NHS) Outcomes Framework for 2013/4 established the DDR as a quality indicator.^3^ Subsequently, NHS England set a national ambition that two‐thirds of all people living with dementia would have a diagnosis; this was translated into a DDR with a numerical value of 66.7%.^4^ This was justified by a timely diagnosis of dementia, occurring when the patient and their family are ready to access help, providing an explanation for symptoms and enabling patients and carers to access post‐diagnostic support, care planning, and treatment, with consequent improvement in health and care outcomes. The policy has recently been confirmed in the NHS 2023/2024 Operating Guidance.^5^

It should be noted that the economic and societal benefits of dementia diagnosis are largely anecdotal and evidence of reductions in institutionalisation and mortality resulting from receiving a dementia diagnosis is lacking.^6^ Nevertheless, the overall benefits of an individual diagnosis, when linked to a meaningful care plan, are overwhelmingly accepted, in line with other areas of clinical practice and are endorsed by the major dementia charities and advocacy organisations.^7^ A diagnosis of dementia is associated with advantages in terms of quality of life for both patients and caregivers. In this context, our focus should be directed towards enhancing the overall quality of life.^8^

**FIGURE 1:**
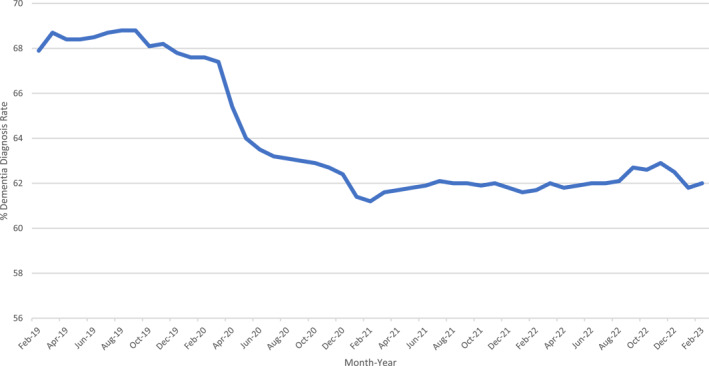
Dementia diagnosis rate (DDR) (%) in people over the age of 65 years between February 2019 and February 2023 in England^12^.

The COVID‐19 pandemic had dramatic consequences for the day‐to‐day running of all health services, including NHS memory services, and a led to a reduction in routine activity.^9^ There was a notable decline in referrals and presentations to nearly all mental and physical health services. This decline can be attributed to both reductions in available service provision and a reduction in patient demand.^10^ While GP referrals from Primary Care to memory services in England have since returned to pre‐pandemic rates, the DDR fell from 67.4% in March 2020 to 62% in February 2023, a reduction of 5.4%^11^, ^12^ shown in Figure 1. It is not clear whether this reduction in the DDR represents a true decrease in diagnostic activity or an artefact of the way that the NHS measures the number of people with dementia in England. We describe potential factors that can impact the national DDR estimates and discuss whether these may have contributed to a lower DDR during the COVID‐19 pandemic.

## 2 ESTIMATED DEMENTIA DIAGNOSIS RATE (DDR)

The national estimated DDR for England is reported monthly by NHS Digital.^12^ The DDR is calculated by dividing the number of people aged over 65 years who have a recorded diagnosis of dementia (from any cause) in each health service region of England, recorded on dementia registers in GP practices in accordance with the Quality and Outcomes Framework guidance (numerator), by the estimated number of people aged over 65 years expected to have dementia in the local population using age‐ and sex‐specific prevalence rates (denominator), multiplied by 100.^13^ Since March 2015, 65+ age and sex specific prevalence rates have been taken from the Cognitive Function and Ageing Study.^14^ CFAS II, conducted in 2011, investigated the prevalence of dementia in people aged 65 or over, and produced age and sex‐specific prevalence rates. The prevalence rates of dementia in CFAS II are lower than those estimated in the previous CFAS I study, conducted in 1993.^14^ The calculation underpinning the DDR has always been debated as there are difficulties in estimating the expected number of people with dementia in the population.^15^

There is variability in diagnosis rates between the seven health regions of England and individual GP clusters. Regional variation in national measures occurs across medical and social care. The January 2023 data show a variation in DDR between five regions of England, ranging from 57.3% to 67.2%.^11^ For context, in the two years prior, between February 2017 and January 2019, the mean DDR was 67.9 (CI 67.8–68.0).^16^ The reported 5.4% point reduction in the national DDR between March 2020 and February 2023 implies that more than 33,000 additional people with dementia are living without a formal diagnosis.^9^ There are, however, other potential explanations which we discuss below.

The numerator in the DDR calculation relies on monthly data collected from GP practices in England on the number of registered patients with a recorded dementia diagnosis. This figure can be impacted by several factors.

### 2.1 Preferral rates to memory services

An impact of the COVID‐19 pandemic was a reduction in GP referral rates. GPs conduct an initial assessment and refer where appropriate to the local memory service for further review. A backlog in referrals might have impacted the DDR.

**FIGURE 2:**
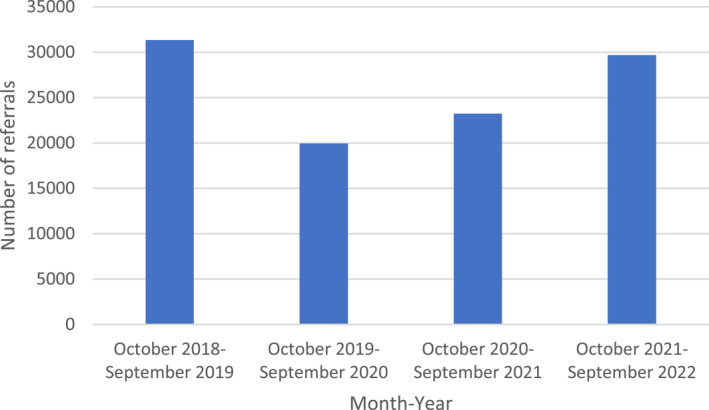
General practitioner referrals to memory services in England between October 2018 and September 2022, by year. Referral data from April ‐ October 2022 is awaiting publication, therefore annual referral cycles October to October are shown.

Although referral rates to memory services dropped sharply in 2020 to only 58% of the number of referrals made in 2019, these have increased again since 2021. At the time of writing, the most recent published data on GP referral rates to memory services in England for September 2022 reveals that the referral rate has returned almost to pre‐pandemic levels (Figure 2).^12^ If the proportion of patients referred by GPs to memory services who ultimately have dementia is lower for example, due to GPs referring more people with functional cognitive symptoms, “brain fog” caused by long COVID^17^, ^18^ and other non‐dementia conditions, this could explain a persistently low DDR despite a recovery in referral rates. However, the proportion of patients referred to memory services receiving a dementia diagnosis was 70% in the 2021 National Audit of Dementia ‐ Memory Services spotlight audit compared to 64% in the 2019 National Memory Service audit, suggesting that the pandemic has not resulted in a rise in non‐dementia referrals.^19^, ^20^ Thus, GP referral rates to memory services are unlikely to be a primary explanation for the DDR not yet recovering to pre‐pandemic levels, assuming that the backlog of referrals delayed due to the pandemic has been eliminated. However, if this referral backlog has remained, there may be an increased number of people awaiting assessment on memory service waiting lists, leading to delayed dementia diagnoses.

### 2.2 Coding of dementia diagnoses

The DDR numerator is reliant on GPs recording the number of people registered with dementia in their care. Dementia diagnoses are recorded by extracting data codes for patients registered with general practices. These codes provide a hierarchical terminology system to encode diagnostic patient information.^21^ GPs are required to code the diagnosis whether made in primary care or communicated by secondary or tertiary care. It may be that diminished routine activity during the COVID‐19 pandemic resulted in a reduction in the imputation of dementia diagnosis codes by GP practices. This is in the context of increasing primary care clinician shortages which may be impacting coding capabilities.^22^ Therefore, even though referral rates were gradually recovering, the coding of dementia diagnoses could have lagged. This could also contribute to the reported drop in the estimated DDR.

Further, the coding system which uses “Read codes” was replaced by Systematized Nomenclature of Medicine Clinical Terms “SNOMED CT codes” in the UK in 2020^23^ with the intent of providing improved, consistent and clearer diagnostic coding.^24^ The transition to the new coding system may have reduced the percentage of patients coded with a dementia diagnosis. This transition coincided with the decline in dementia diagnosis rates and may have been a contributing factor. To illustrate this, in 2013 there was a major drive to improve the coding of dementia diagnoses in London general practices which was thought to be contributing to low rates of dementia diagnosis on practice registers. This initiative resulted in an increase in the London DDR, with the proportion of (borough‐based) London Clinical Commissioning Groups achieving rates of at least 66.7% increasing from 46% in 2013 to 84% by 2017.^25^

### 2.3 Mild cognitive impairment diagnoses and the national dementia audits

One potential reason for the drop in DDR during the COVID‐19 pandemic could be memory service practitioners diagnosing patients with mild cognitive impairment (MCI) rather than dementia, potentially reflecting more diagnostic uncertainty in the context of poorer quality assessments taking place remotely over the telephone or via video. For example, in 2021, 35% of patients had a virtual appointment.^19^ However, available memory service audit data do not support this concern and available survey data collected from patients after virtual memory service assessments were positive.^26^ Two national memory service audits in the UK were conducted in 2019 and 2021.^19^, ^27^ The 2019 National Memory Service audit was conducted by NHS England and included 85 memory services with data collected on 3978 patients.^20^ The 2021 National Audit of Dementia ‐ Memory Services spotlight audit included 63 memory services with data collected on 5970 patients in England and Wales.^19^ A breakdown of the proportion of diagnoses made in the audit data is provided in Table 1, which shows that the rate of MCI diagnoses has actually decreased between the 2019 and 2021 audits, with a corresponding 6% increase in dementia diagnoses.

**TABLE 1:** Percentage diagnoses of dementia, mild cognitive impairment and other in the 2019 National Memory Service audit and the 2021 National Audit of Dementia—Memory Services spotlight audit.

Audit year	Dementia	Mild cognitive impairment	Other
2019	64%	17%	19%
2021	70%	16%	14%

*Note*: Data is separate to national data published in NHS Digital and the regional data from South Staffordshire memory service.

### 2.4 Local memory service data

**FIGURE 3:**
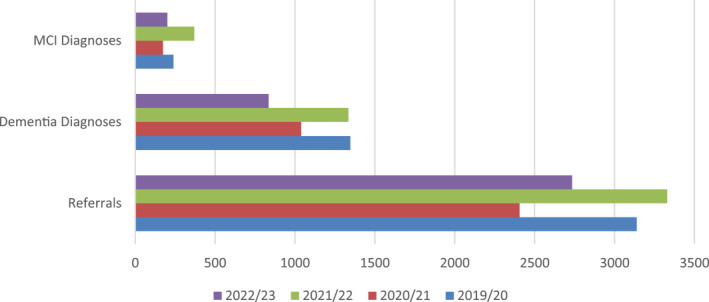
Number of memory clinic referrals, dementia diagnoses and mild cognitive impairment (MCI) diagnoses made between 2019 and 2023 to the South Staffordshire memory service, by year from April‐March. The 2022/23 data is from April‐December.

Data were analysed at the local memory service level from South Staffordshire memory service, which is operated by Midlands Partnership NHS Foundation Trust (Figure 3). This provided data about rates of MCI diagnoses which is not collected at national level and is an alternative source to GP registrations. Data are provided by year from April‐March. The 2022/23 data are an incomplete dataset, covering April‐December. The local trend reveals a reduction in memory clinic referrals, and dementia and MCI diagnoses in 2020/2021. In 2021/2022 the rates of referrals and diagnoses returned to pre‐pandemic levels and in fact, exceeded those from 2019/2020. This may support the argument that the lower DDR was in part due to the backlog of cases due to the pandemic. This data, albeit from only one memory service, provides evidence that the number of dementia diagnoses has returned to pre‐pandemic levels, contrary to the national data.

### 2.5 Excess dementia deaths due to COVID‐19

So far we have considered factors affecting the DDR numerator alone. We must also consider whether an apparent fall in DDR might be an artefactual error due to reduced accuracy of the denominator. The true prevalence of dementia in the population is a function of dementia incidence and mortality rates.^14^ During the COVID‐19 pandemic, people with dementia experienced a 25% higher risk of dying during the pandemic period compared to previous years.^28^ Over a quarter (27.5%) of those who died from COVID‐19 in England and Wales between March to June 2020 had a diagnosis of dementia, equivalent to 13,840 deaths.^29^ As a result, dementia was the most common pre‐existing condition in deaths involving COVID‐19 in England and Wales during this period.^30^ The total number of people aged over 65 years on GP dementia registers in England decreased from 437,882 to 415,778 between March 2020 and March 2021,^11^ which closely aligns with the 21,798 reported excess deaths due to COVID‐19 in people diagnosed with dementia in England over the same period.^31^ People with a dementia diagnosis also accounted for the largest increase in excess non‐COVID‐19 related deaths.^29^ Therefore, during lockdown, people with dementia also had a higher risk of death without confirmed COVID‐19 which would have impacted on mortality rates in this population further.^32^

Therefore, it is likely that the COVID‐19 pandemic has caused a decline in the prevalence of dementia, such that estimates based on CFAS II are no longer accurate. This would lead to a DDR denominator that is higher than the true prevalence of dementia, which (given the decline in the numerator due to excess mortality) would lead to an apparent reduction of the DDR. In other words, the observed DDR decrease during the pandemic may be driven by a reduction in dementia prevalence due to the disproportionately higher death rate affecting individuals with dementia during the COVID‐19 pandemic. This could be elucidated by looking at the stage of dementia of patients on the GP register; deaths from COVID‐19 were commoner in people in the later stages of dementia whereas “new” additions tend to be people in the earlier stages.^33^ However, GP dementia registers do not record dementia severity.

## 3 CONCLUSION

The COVID‐19 pandemic had a large impact on the activity of primary care and dementia‐related secondary care services in England, with an acute reduction in referrals and diagnoses. An increase in cases of undiagnosed dementia may be a byproduct of extended waiting periods for diagnosis, and this would be an important area of further study. At the time of writing, the estimated DDR has not returned to pre‐pandemic levels but there are recent signs of a recovery.^11^ In this paper we have explored possible reasons for this, including a reduction in diagnostic code imputation or, of concern to those for policymakers, a potential decrease in true dementia prevalence secondary to excess COVID‐19 deaths which has yet to be reflected in the DDR denominator. It would also be useful to compare dementia diagnoses rates in England during the COVID‐29 pandemic with global data. For example, in Sweden there was a larger decline in dementia diagnoses during 2020 which was not correlated with mortality.^34^

To understand the landscape of dementia diagnoses in England it is imperative that the prevalence rates of dementia are accurately captured. An up‐to‐date multicentre population‐based cohort study is required to understand any changes in the epidemiology of dementia in the wake of the COVID‐19 pandemic. This would help to inform policymakers whether further increases in England's DDR for example, through improved recognition of dementia, access to memory services and investigations, and training of memory service clinicians, is a priority. It is of note that the DDR does not currently inform on the NHS's performance in diagnosing young‐onset dementia,^35^ as this population was not included in CFAS II (although the total number of people aged under 65 with dementia is published alongside the DDR. Furthermore, a more granular understanding of dementia prevalence at local level, in which the DDR denominator is adjusted for factors known to affect dementia susceptibility such as deprivation, rurality, and ethnicity will also increase the utility of the DDR as an instrument which guides public policy.^36^, ^37^, ^38^, ^39^, ^40^, ^41^, ^42^ Nevertheless, the DDR (perhaps because of its form—a simple percentage rate accessible to all) has maintained the profile of access to diagnosis, an essential element in the dementia pathway.

## CONFLICT OF INTEREST STATEMENT

AB is the National Clinical Director for Dementia at NHS England (he is a co‐author in his academic capacity). JDI is Clinical Director of NHS England (London) dementia clinical network. He has received conference expenses and consultancy fees (paid to his institution) from Roche, a speaker's fee (paid to his institution) from Biogen and payment (to his institution) from Nestle Health Science for membership of a clinical trial steering committee. The other authors have no conflicts of interest to declare.

## Data Availability

Data sharing not applicable to this article as no datasets were generated or analysed during the current study.

## References

[1] Prince M , Knapp M , Guerchet M , et al. Dementia UK: ‐Overview; 2014. Published online.

[2] Department of Health . Prime Minister’s Challenge on Dementia 2020; 2015. https://assets.publishing.service.gov.uk/government/uploads/system/uploads/attachment_data/file/414344/pm‐dementia2020.pdf

[3] Department of Health . The NHS Outcomes Framework 2012/13; 2011. https://assets.publishing.service.gov.uk/government/uploads/system/uploads/attachment_data/file/213711/dh_131723.pdf

[4] NHS England . Dementia; 2021. Published . Accessed 14 February 2023. https://www.england.nhs.uk/mental‐health/dementia/

[5] NHS England . 2023/24 Priorities and Operational Planning Guidance. NHS England; 2023. Accessed 21 March 2023. https://www.england.nhs.uk/wp‐content/uploads/2022/12/PRN00021‐23‐24‐priorities‐and‐operational‐planning‐guidance‐v1.1.pdf

[6] Aldus C , Arthur A , Dennington‐Price A , et al. Undiagnosed dementia in primary care: a record linkage study. Health Serv Deliv Res. 2020;8(20):1‐108. 10.3310/hsdr08200 32310343

[7] Alzheimer’s Society . Why Is it Important to Get Dementia Diagnosed? 2022. Published. Accessed March 20, 2023. https://www.alzheimers.org.uk/about‐dementia/symptoms‐and‐diagnosis/dementia‐diagnosis/why‐get‐dementia‐diagnosed

[8] Gomes M , Pennington M , Wittenberg R , Knapp M , Black N , Smith S . Cost‐effectiveness of Memory Assessment Services for the diagnosis and early support of patients with dementia in England. J Health Serv Res Pol. 2017;22(4):226‐235. 10.1177/1355819617714816 28622732

[9] NHS0019 Written Evidence Submitted by the Alzheimer’s Society. The Alzheimer’s Society. 2021.

[10] Chen S , Jones PB , Underwood BR , et al. The early impact of COVID‐19 on mental health and community physical health services and their patients’ mortality in Cambridgeshire and Peterborough, UK. J Psychiatr Res. 2020;131:244‐254. 10.1016/j.jpsychires.2020.09.020 33035957 PMC7508053

[11] NHS Digital . Primary Care Dementia Data; 2023. Published March 16, 2023. Accessed March 20, 2023. https://digital.nhs.uk/data‐and‐information/publications/statistical/primary‐care‐dementia‐data/february‐2023

[12] NHS Digital . Series/Collection Primary Care Dementia Data; 2023. Published . Accessed 14 February 2023. https://digital.nhs.uk/data‐and‐information/publications/statistical/primary‐care‐dementia‐data

[13] NHS Digital . Recorded Dementia Diagnoses ‐ Methodology of Indicators; 2022. Published July 11. Accessed 14 February 2023. https://digital.nhs.uk/data‐and‐information/publications/statistical/recorded‐dementia‐diagnoses/recorded‐dementia‐diagnoses‐supporting‐information/recorded‐dementia‐diagnoses‐methodology#detailed‐description

[14] Matthews FE , Arthur A , Barnes LE , et al. A two‐decade comparison of prevalence of dementia in individuals aged 65 years and older from three geographical areas of England: results of the Cognitive Function and Ageing Study I and II. Lancet. 2013;382(9902):1405‐1412. 10.1016/s0140-6736(13)61570-6 23871492 PMC3906607

[15] Donegan K , Fox N , Black N , Livingston G , Banerjee S , Burns A . Trends in diagnosis and treatment for people with dementia in the UK from 2005 to 2015: a longitudinal retrospective cohort study. Lancet Public Health. 2017;2(3):e149‐e156. 10.1016/s2468-2667(17)30031-2 29253388

[16] NHS Digital . Recorded Dementia Diagnoses; 2022. Published October 20. Accessed 26 June 2023. https://digital.nhs.uk/data‐and‐information/publications/statistical/recorded‐dementia‐diagnoses

[17] Perlis RH , Santillana M , Ognyanova K , et al. Prevalence and correlates of long COVID symptoms among US adults. JAMA Netw Open. 2022;5(10):e2238804. 10.1001/jamanetworkopen.2022.38804 36301542 PMC9614581

[18] Ball HA , McWhirter L , Ballard C , et al. Functional cognitive disorder: dementia’s blind spot. Brain. 2020;143(10):2895‐2903. 10.1093/brain/awaa224 32791521 PMC7586080

[19] National Audit of Dementia Memory Assessment Services Spotlight Audit 2021. Royal College of Psychiatrists; 2022. Accessed February 14, 2023. www.nationalauditofdementia.org.uk

[20] Cook L , Souris H , Isaacs J . The 2019 National Memory Service Audit. Published online March 2020. Accessed 13 March 2021. https://www.england.nhs.uk/london/wp‐content/uploads/sites/8/2020/04/The‐2019‐national‐memory‐service‐audit.pdf

[21] Benson T . The history of the read codes: the inaugural James read memorial lecture 2011. Inf Prim Care. 2011;19(3):173‐182. 10.14236/jhi.v19i3.811 22688227

[22] Nussbaum C , Massou E , Fisher R , Morciano M , Harmer R , Ford J . Inequalities in the distribution of the general practice workforce in England: a practice‐level longitudinal analysis. BJGP open. 2021;5(5). 10.3399/bjgpo.2021.0066 PMC8596307 34404634

[23] Tulloch JS , Beadsworth MB , Vivancos R , Radford AD , Warner JC , Christley RM . GP coding behaviour for non‐specific clinical presentations: a pilot study. BJGP open. 2020;4(3):bjgpopen20X101050. 10.3399/bjgpopen20x101050 PMC7465576 32636202

[24] Millar J . The need for a global language‐SNOMED CT introduction. Stud Health Technol Inf. 2016;225:683‐685. 27332304

[25] Cook L , Harwood D . Improving Dementia Diagnosis Rates in London; 2019. Accessed 14 February 2023. https://www.england.nhs.uk/london/wp‐content/uploads/sites/8/2019/07/dem‐improving‐diagnosis‐poster.pdf

[26] Aftab A , Sidhom E , Forrest A , et al. Patient and clinician experience of providing remote memory assessment services. Int J Geriatr Psychiatr. 2022;37(2). 10.1002/gps.5664 34866239

[27] Cook LD , Nichol KE , Isaacs JD . The London memory service audit and quality improvement programme. BJPsych Bull. 2019;43(5):215‐220. 10.1192/bjb.2019.18 PMC12402916 30898180

[28] Liu KY , Howard R , Banerjee S , et al. Dementia wellbeing and COVID‐19: review and expert consensus on current research and knowledge gaps. Int J Geriatr Psychiatr. 2021;36(11):1597‐1639. 10.1002/gps.5567 PMC8237017 34043836

[29] Alzheimer’s Society . Worst Hit: Dementia during Coronavirus; 2021. Accessed 14 February 2023. https://www.alzheimers.org.uk/sites/default/files/2020‐09/Worst‐hit‐Dementia‐during‐coronavirusreport.%20pdf

[30] Burns A , Howard R . COVID‐19 and dementia: a deadly combination. Int J Geriatr Psychiatr. 2021;36(7):1120‐1121. 10.1002/gps.5551 PMC8206864 33961712

[31] Office for Health Improvement and Disparities . Excess Mortality in England.

[32] Chen S , Jones PB , Underwood BR , et al. Risk factors for excess deaths during lockdown among older users of secondary care mental health services without confirmed COVID‐19: a retrospective cohort study. Int J Geriatr Psychiatr. 2021;36(12):1899‐1907. 10.1002/gps.5610 PMC8420159 34382242

[33] Matias‐Guiu JA , Pytel V , Matías‐Guiu J . Death rate due to COVID‐19 in Alzheimer’s disease and frontotemporal dementia. J Alzheim Dis. 2020;78(2):537‐541. 10.3233/jad-200940 33074240

[34] Axenhus M , Frederiksen KS , Zhou RZ , Waldemar G , Winblad B . The impact of the COVID‐19 pandemic on mortality in people with dementia without COVID‐19: a systematic review and meta‐analysis. BMC Geriatr. 2022;22(1):1‐9. 10.1186/s12877-022-03602-6 36402953 PMC9675075

[35] Cook LD , Souris H , Isaacs JD . Differences in care between younger and older patients in the 2019 English national memory service audit. BJPsych Bull. 2022;46(6):315‐321. 10.1192/bjb.2021.104 34782030 PMC9813762

[36] Russ TC , Batty GD , Hearnshaw GF , Fenton C , Starr JM . Geographical variation in dementia: systematic review with meta‐analysis. Int J Epidemiol. 2012;41(4):1012‐1032. 10.1093/ije/dys103 22798662 PMC3429875

[37] Cassarino M , O’Sullivan V , Kenny RA , Setti A . Environment and cognitive aging: a cross‐sectional study of place of residence and cognitive performance in the Irish longitudinal study on aging. Neuropsychology. 2016;30(5):543‐557. 10.1037/neu0000253 26595827

[38] Hofbauer L , Rodriguez F . The role of social deprivation and depression in dementia risk: findings from the longitudinal survey of health, ageing and retirement in Europe. Epidemiol Psychiatr Sci. 2023;32:e10. 10.1017/s2045796023000033 36786038 PMC9971857

[39] Chung S , Providencia R , Sofat R , et al. Incidence, morbidity, mortality and disparities in dementia: a population linked electronic health records study of 4.3 million individuals. Alzheimer’s & Dementia. 2023;19(1):123‐135. 10.1002/alz.12635 PMC10078672 35290719

[40] Jitlal M , Amirthalingam GN , Karania T , et al. The influence of socioeconomic deprivation on dementia mortality, age at death, and quality of diagnosis: a Nationwide death records study in England and Wales 2001–2017. J Alzheim Dis. 2021;81(1):321‐328. 10.3233/jad-210089 33780372

[41] Pham TM , Petersen I , Walters K , et al. Trends in dementia diagnosis rates in UK ethnic groups: analysis of UK primary care data. Clin Epidemiol. 2018;10:949‐960. Published online. 10.2147/clep.s152647 30123007 PMC6087031

[42] Mukadam N , Marston L , Lewis G , Mathur R , Rait G , Livingston G . Incidence, age at diagnosis and survival with dementia across ethnic groups in England: a longitudinal study using electronic health records. Alzheimer’s & Dementia. 2022;19(4):1300‐1307. Published online. 10.1002/alz.12774 36047605

